# Predictive Model for Mortality Risk Stratification Using Laboratory Biomarkers in Singaporeans

**DOI:** 10.1093/geroni/igaf122.3829

**Published:** 2025-12-31

**Authors:** Xinru Lim, Jiangfeng Ye, Denise Goh, Jess Vo, Roger Ho, Min-Han Tan, Joe Yeong

**Affiliations:** Institute of Molecular and Cell Biology, Singapore, Singapore; National University of Singapore, Singapore, Singapore; Institute of Molecular and Cell Biology, Singapore, Singapore; Lucence Diagnostics Pte. Ltd., Singapore, Singapore; National University of Singapore, Singapore, Singapore; Lucence Diagnostics Pte. Ltd., Singapore, Singapore; Institute of Molecular and Cell Biology, Singapore, Singapore

## Abstract

Current clinical decision-making tools often rely on aggregate scoring systems or subjective assessments, which may not capture early or subtle physiological changes indicating high mortality risk. While laboratory tests are ubiquitously available and inexpensive, their prognostic potential is underutilized. There is a need for an evidence-based, quantitative tool that can harness the predictive power of routine laboratory data to improve patient outcomes by identifying high-risk individuals earlier and more accurately. With two independent cohorts of older Asians, aged ≥55 years, from the Singapore Longitudinal Ageing Studies, we investigated their basic clinical laboratory biomarkers (i.e. full blood count and biochemistry variables) and its relation to all-cause mortality. Based on risk score derived from a Cox model trained to predict mortality risk, we identified a group of individuals with significantly shorter overall survival (P < 0.01), using 17 years of follow-up data. Subsequent Cox Regression analysis demonstrated robust predictive performance for 5-year and 10-year mortality. This result was also validated in the National Health and Nutrition Examination Survey cohort, achieving a concordance index of 0.79 and 0.78, respectively (for aged ≥40 years). Furthermore, we revealed that the biological age was significantly elevated in deceased individuals compared to their chronological age (P < 0.0001), while surviving individuals exhibited a younger biological age relative to their chronological age (P < 0.0001). Consequently, we developed a predictive model that utilizes routinely analyzed laboratory biomarkers-including components of the immune cell count and other clinical variables-to assess and stratify mortality risk, capable of identifying high-risk individuals earlier and more accurately.

